# Effects of co-incubation of LPS-stimulated RAW 264.7 macrophages on leptin production by 3T3-L1 adipocytes: a method for co-incubating distinct adipose tissue cell lines

**DOI:** 10.1186/s42269-022-00747-7

**Published:** 2022-03-07

**Authors:** Cristina Caldari-Torres, Jordan Beck

**Affiliations:** Department of Biology, Denison University, Granville, OH USA

**Keywords:** Macrophages, Adipocytes, Inflammation, IL-6, Leptin

## Abstract

**Background:**

Adipose tissue is a major endocrine organ capable of releasing inflammatory adipokines that are linked to changes occurring in the overfed state, where tissue remodeling results in hypertrophic adipocytes that recruit monocytes to infiltrate the tissue and take on an inflammatory phenotype. Increases in macrophage-specific inflammatory mediator levels contribute to the inflamed state and worsen the inflammatory loop between the macrophages and adipocytes. Although most inflammatory adipokines are released by macrophages, adipocytes can also release immunomodulatory adipokines, such as leptin. The objective of this research was to determine if co-incubation of activated macrophages with mature adipocytes, using transwell inserts, affected adipocyte leptin release. We also examined if there were differences in levels of cell-secreted products quantified in cell-conditioned media collected from macrophage-containing (transwell insert) and adipocyte-containing (well) compartments.

**Methods:**

Mature adipocytes were co-incubated with control and lipopolysaccharide-stimulated (0.01 mg/ml) murine macrophages, and nitric oxide, interleukin-6, and leptin levels were quantified in the cell-conditioned media from both compartments.

**Results:**

Activation status of the macrophages did not affect leptin release by the adipocytes. We observed higher amounts of leptin in wells compared to transwells. Nitric oxide and interleukin-6 levels were similar between transwells and wells, suggesting that these adipokines travel through the transwell inserts and are reaching equilibrium between the two compartments.

**Conclusion:**

Our results suggest that co-incubating activated macrophages and adipocytes using transwell inserts can result in distinct microenvironments in the different cellular compartments and that separate sampling of these compartments is required to detect the subtle signaling dynamics that exist between these cells.

**Supplementary Information:**

The online version contains supplementary material available at 10.1186/s42269-022-00747-7.

## Background

Adipose tissue (AT) has emerged as a major endocrine organ capable of releasing inflammatory mediators, adipokines, that may result in a chronic inflammatory state. Low levels of chronic inflammation, or meta-inflammation, may predispose the individual to chronic disease, including insulin resistance and type 2 diabetes mellitus (Ouchi et al. 2011; Sorisky et al. 2013) and is currently implicated in adverse COVID-19 outcomes (Dalamaga et al. 2021). Adipokines are AT-derived proteins, a category that includes, but is not limited to, cytokines, chemokines, and hormones. In addition to adipocytes, AT contains adipocyte precursors in various stages of differentiation, as well as non-fat cells like endothelial and immune cells. Among the immune cells present in AT, macrophages have received a lot of attention by adipoimmunologists due to their central role in AT-derived inflammation (Vieira-Potter 2014; Russo and Lumeng 2018; Monteiro et al. 2019).

Macrophages are innate immune cells that circulate as monocytes and differentiate into their final phenotype based on the microenvironment encountered in the tissue they extravasate into for residence. A comprehensive review of adipokine release from AT concluded that non-fat cells, mostly macrophages, release the majority of the inflammatory adipokines that are increased in the obese state (Fain 2010). Some of the adipokines found at higher circulating levels in obese individuals include interleukin (IL)-6, IL-8, tumor necrosis factor (TNF)-α, monocyte chemoattractant protein (MCP)-1, and leptin (Fain 2010). Of these adipokines, leptin is the only one that is primarily produced by the adipocytes (Fain 2010). Leptin is positively correlated with obesity and has a major role in regulation of energy homeostasis through its interactions with neural systems that regulate energy stores, specifically through its role in establishing the adiposity set point (Friedman and Halaas 1998; (LeDuc et al. 2021). Leptin also has effects on both the innate and adaptive branches of the immune system. With regards to monocytes/macrophages, which express the functional leptin receptor (Loffreda et al. 1998; Gelsomino et al. 2020; Pérez-Pérez et al. 2020), this adipokine has been implicated in modulation of cytokine production and phagocytosis (Pérez-Pérez et al. 2020), as well as differentiation of monocytes into pro-inflammatory M1 macrophages (Acedo et al. 2013; Santos-Alvarez et al. 1999; Becerril et al. 2019). Unno et al. (2006) reported that nitric oxide (NO), a signaling molecule that is readily produced by activated macrophages, downregulated leptin expression at both the protein and mRNA level in murine adipocytes (3T3-L1 cell line). The NO used in the study was derived from various NO donors (NOC7, NOC18, and GSNO) and autocrinally from the adipocytes. There are currently no reports on the effects of macrophage-derived adipokines, including NO, on leptin production by adipocytes (Becerril et al. 2019).

The inflamed microenvironment caused by AT-derived adipokines in the obese state drives immune cell recruitment (Bai and Sun 2015; Fain 2010; Liu and Nikolajczyk 2019), increasing the percentage of macrophages residing in obese compared to lean AT. In lean AT, macrophages make up ~ 10% of cells (Osborn and Olefsky 2012) and mostly exhibit the alternatively activated (M2) anti-inflammatory phenotype. M2 macrophages have housekeeping functions ranging from immune surveillance to clearance of cellular debris and lipid buffering (Boutens and Stienstra 2016). In obese AT, there is an increase in total macrophage numbers (making up to 50–60% of AT) as well as in the number of macrophages exhibiting a classically activated or M1, pro-inflammatory phenotype (Osborn and Olefsky 2012; Liu and Nikolajczyk 2019). Pro-inflammatory M1 macrophages express higher levels of TNF-α and inducible nitric oxide synthase (iNOS) (Lumeng et al. 2007). Using DNA microarray gene analyses, Yamashita et al. (2008) concluded that low levels (1 ng/ml) of bacterial lipopolysaccharide (LPS) drive RAW264.7 murine macrophages to differentiate into M1 macrophages, increasing expression of cyclooxygenase-2, iNOS, TNF-α, and activation of NF-κB. Factors that increase monocyte recruitment to obese AT and their differentiation into the M1 macrophage phenotype include hypertrophy of adipocytes, which coupled with hypoxia and oxidative stress (Lindhorst et al. 2021) lead to inflammatory adipokines and chemokines release by AT and increased adipocyte apoptosis (Arner et al. 2012; Patel and Abate 2013; Lindhorst et al. 2021). Adipocyte survival and maturation/differentiation is affected by increases in M1 macrophage numbers in AT, resulting in a decrease in adipocyte hyperplasia, the production of new adipocytes, during times of chronic positive energy balance, and instead favoring hypertrophy of already existing adipocytes (Sorisky et al. 2013). Hypertrophic adipocytes are associated with augmented inflammation and dysfunctional insulin sensitivity (Heilbronn et al. 2004). Inflammatory adipokines released by M1 macrophages, like TNF-α and IL-6, can block insulin action in adipocytes via autocrine/paracrine mechanisms (Makki et al. 2013), linking the increased macrophage recruitment and M1 polarization observed in obese AT with impaired insulin sensitivity. These inflammatory adipokines also result in adipocyte mitochondrial dysregulation, through an increased release of reactive oxygen species and mitochondrial fragmentation, adding a further layer of complexity to the adipocyte-macrophage cross-talk and potentiation of inflammation in obese adipose tissue (Vieira-Potter 2014).

Increased energy storage associated with obesity causes hypertrophic, hypoxic, and apoptotic adipocytes that release increasing amounts of inflammatory adipokines. The inflamed microenvironment favors recruitment of macrophages toward obese AT and polarizes them toward the M1 inflammatory phenotype which, in turn, release macrophage-specific inflammatory adipokines that further support adipocyte hypertrophy and recruitment of monocytes from circulation, creating an inflammatory loop. The cross-talk between macrophages and adipocytes and their precursors is central to AT-derived inflammation, as it maintains the inflammatory loop and aids in the recruitment of new macrophages that will likely develop an M1 phenotype (Bai and Sun 2015; Pérez-Pérez et al. 2020).

The main objective of this research was to determine the effects of co-incubating murine 3T3-L1 adipocytes and activated RAW264.7 macrophages on the production of two inflammatory adipokines, IL-6 and leptin, by these two cell types. Specifically, we wanted to test if the activation status of the macrophages would exert paracrine effects on the mature adipocytes, as measured by secretion of leptin, an adipocyte-specific adipokine. These objectives were tested through the use of transwell inserts (0.4 μm pore size), which allow for the co-incubation of different cell lines and exposure of one cell line to products secreted by the other cell line. We also examined if there was a difference in the amount of cell-secreted products quantified in the cell-conditioned media collected from macrophage-containing transwells and adipocyte-containing wells. Sampling each cell types’ microenvironment would allow us to detect the potential subtle signaling dynamics that exist between these cells.

## Methods

### Reagents and materials

Murine fibroblast (3T3-L1, cat no. CL-173) and macrophage (RAW 264.7, cat no. TIB-71) immortalized cell lines were purchased from ATCC (Manassass, VA). Dulbecco’s modified Eagle’s medium (DMEM), phosphate buffered saline (PBS), fetal bovine serum (FBS), penicillin–streptomycin, and polystyrene 6-well plates were purchased from Fisher Scientific (Pittsburg, PA). The IL-6 (DY406-05-Duo Set for preliminary experiments and SM6000B-quantikine for all other experiments) and leptin (SMOB00B) enzyme-linked immunosorbent assays (ELISA) were purchased from R&D systems (Minneapolis, MN), while the Griess assay for nitric oxide (NO) quantification was obtained from Promega (Madison, WI). Trypan blue, insulin, dexamethasone (DEX), d-biotin, 3-isobutyl-1-methylxanthine (IBMX), trypsin–EDTA 0.25%, oil red O dye, and lipopolysaccharide (LPS) were purchased from Sigma-Aldrich (St Louis, MO). Transwell permeable supports (0.4 μm pore size, 12 mm diameter, polyester membrane) and 12-well plates (polystyrene) were obtained from Corning Costar (Corning, NY).

### 3T3-L1 cell maintenance, culture, and differentiation into mature adipocytes

The 3T3-L1 cell line must be differentiated from a fibroblast phenotype into its final, mature adipocyte phenotype containing lipid droplets. To do this, the cells were incubated in 12-well plates, in a 5% CO_2_ humidified atmosphere, and were kept in the undifferentiated fibroblast phenotype at less than 50% confluency during sub-culturing. Detachment of cell monolayer for sub-culturing was performed via trypsinization. Growth medium for 3T3-L1 cells consisted of DMEM, 10% (v/v) heat-inactivated FBS, 1% antibiotics (100 U/ml penicillin and 100 μg/ml streptomycin), and 0.008 μg/ml D-biotin. Differentiation into the adipocyte phenotype was performed as described by Zebisch et al. (2012). Briefly, three days after cells reached ~ 90% confluency and started to clump together and lose fibroblast morphology, they were washed with 1X PBS and treated with a differentiation cocktail consisting of growth medium supplemented with 0.5 mM IBMX, 1 μM DEX, and 20 μg/ml insulin. Forty-eight hours after addition of the differentiation cocktail, the cells were washed with 1X PBS and treated with post-differentiation medium consisting of growth medium supplemented with 20 μg/ml insulin. Treatment with post-differentiation medium was performed every forty-eight hours for a total of four times. At the end of the differentiation period, lipid droplets inside the adipocytes could be visualized using an inverted microscope. In preliminary experiments, lipid droplet deposition was quantified through spectrophotometric analysis of Oil Red O staining as described by Manickam et al. (2010) to confirm the differentiation of the fibroblasts into the mature adipocyte phenotype. Briefly, both differentiated and undifferentiated cells were washed with cold PBS (pH 7.4) and subsequently fixed with a 4% paraformaldehyde solution. The cells were then stained for 30 min with Oil Red O dye (0.5% w/v) prepared in isopropanol and diluted to a working solution at a 3:2 ratio of dye/water. Cells were then washed thoroughly with water, and then, the dye in the lipid droplets was dissolved with isopropanol. The isopropanol-dye solution was analyzed spectrophotometrically at A_520 nm_, and values obtained for undifferentiated cells were compared to those of differentiated cells (Additional file 1: Fig. S1).

### RAW 264.7 cell maintenance and culture

RAW 264.7 cells were grown in polystyrene 6-well plates with DMEM supplemented with 10% (v/v) heat-inactivated FBS and 1% antibiotics (100 U/ml penicillin and 100 μg/ml streptomycin). Cells were incubated in a 95% O_2_ and 5% CO_2_ humidified atmosphere. During initial expansion, the medium was changed every two days after washing cells with 1× PBS. Cells were not grown beyond 80% confluency during expansion. Detachment of cell monolayer for sub-culturing was performed with the cell scraping method. When cells reached 80% confluency, they were transferred to transwell inserts to commence the co-incubation experiments.

### RAW 264.7 cell activation

A concentration of 0.01 μg/ml of LPS was used, and three methods of LPS challenge for co-incubation experiments were tested: macrophages in 6-well plate were washed, resuspended (via scraping) in fresh medium, transferred to transwell inserts and challenged with LPS that was added into the transwell compartment (3T3 + RAW + LPS); macrophages were challenged with LPS for 24 h while in well of 6-well plate, were then washed, resuspended (via scraping) in fresh medium, and transferred to transwell insert (3T3 + StimRAW); macrophages were challenged with LPS for 24 h while in well of 6-well plate and then resuspended (via scraping) and transferred to transwell insert along with the conditioned media (3T3 + StimRAW + CondMed). Non-LPS-challenged macrophages were co-incubated with adipocytes as a control (3T3 + RAW). The LPS concentration (0.01 mg/ml) and incubation time (6 h) were selected based on preliminary experiments testing the effects of low LPS doses that resulted in high production of IL-6 and NO by the macrophages (Additional file 1: Fig. S2). The highest IL-6 production was observed when incubating the cells with 0.01 mg/ml of LPS for 6 h (Additional file 1: Fig. S2a). While 0.1 mg/ml of LPS resulted in significantly higher NO production by the macrophages after 6 and 24 h of incubation compared to 0.01 mg/ml (Additional file 1: Fig. S2b), both LPS doses caused the macrophages to produce IL-6 concentrations beyond the limit of detection of the assay (IL-6 DuoSet ELISA). Based on these results, we selected the lowest dose that resulted in a strong response in order to utilize a concentration that falls within the bounds of what is considered physiologically and clinically relevant (Guo et al. 2013). The macrophages did not produce quantifiable levels of leptin under any of the LPS concentrations or incubation times (data not shown).

### Quantification of nitric oxide (NO) production

Macrophage activation was quantified via measurement of NO levels. Nitric oxide production by the macrophages was determined through quantifying nitrite levels in cell-conditioned media using the Griess assay. Briefly, 50 μL of cell-conditioned media was added in triplicate to a 96-well plate and mixed with 50 μL of sulfanilamide solution and allowed to incubate for 10 min. Following the incubation, 50 μL of N-1-naphthylethylenediamine (NED) was added to each well, followed by a 10 min incubation. After the second incubation, absorbance was measured at 530 nm. Nitrite concentrations were determined by extrapolating absorbance measurements from a 0–100 μM standard curve. An Epoch plate reader (BioTek Instruments, Winooski, VT) was used for absorbance measurements (Chen et al. 2017).

### Co-incubation of RAW 264.7 and 3T3-L1 cells using transwell inserts

Several co-incubation methodologies were tested to determine if they resulted in different activation level of the macrophages, as measured by NO and IL-6 levels in cell-conditioned medium. Additionally, leptin levels in cell-conditioned medium were measured to test if activation state of the macrophages affected production of this adipokine by the mature adipocytes. Three methods of macrophage stimulation/co-incubation were tested. Mature adipocytes (differentiated according to the steps described in “3T3-L1 cell maintenance, culture, and differentiation into mature adipocytes” section) in 12-well plates were co-incubated with macrophages stimulated with LPS as described in the “RAW 264.7 cell activation” section: 1. 3T3 + RAW + LPS, 2. 3T3 + StimRAW, and 3. 3T3 + StimRAW + CondMed. Additionally, adipocytes were co-incubated with unstimulated RAW264.7 cells resuspended in fresh medium (3T3 + RAW) as a control. On average, 8.0 × 10^5^ macrophages were plated onto each transwell insert in a total of 500 μl. Twenty-four hours after co-incubation commenced, media was collected separately from the transwell inserts and wells, transferred to 1.5 ml microcentrifuge tubes, and stored at − 20 °C until used for IL-6, leptin, and NO quantification. Wells were run in duplicate, and experiments were performed four times.

### Quantification of adipokine production

Analyses of cell-conditioned media for determination of adipokine levels were done using IL-6 and leptin sandwich ELISAs according to manufacturer’s instructions. Samples were tested in triplicate, and a standard curve was produced and used to extrapolate the cytokine concentrations in the samples. Experimental samples were analyzed using the quantikine IL-6 ELISA, and samples measuring > 500 pg/ml (highest standard) were diluted, re-quantified, and results were adjusted taking the dilution factor into account. Samples from LPS dose/time response preliminary experiments were analyzed using the DuoSet IL-6 ELISA, and samples measuring above the 1000 pg/ml highest standard of the DuoSet IL-6 ELISA were analyzed as “1000 pg/ml.” An Epoch plate reader was used for absorbance measurements.

### Statistical analyses

Statistical analyses were done using JMP Pro 13 (Cary, NC). Non-normal data were normalized using a log transformation. Matched paired *t* tests were used to determine differences in NO, IL-6, and leptin levels between cell-conditioned medium collected from transwell inserts containing macrophages and wells containing adipocytes. In order to determine differences in NO, IL-6, and leptin produced by control and LPS-challenged cells, Student’s *t* tests were used. Linear regression analyses were used to test relationships between IL-6, NO, and leptin. Interleukin-6, leptin, and NO concentrations in cell-conditioned media were analyzed using the general mixed linear model. All statistical analyses were conducted using JMP Pro 15 (SAS, Cary, North Carolina). The sources of variation included experiment, treatment, experiment x treatment interaction, and well nested within experiment x treatment interaction. The experiment, treatment × experiment interaction, and well nested within experiment x treatment interaction were considered as random variables. When treatment effects were detected, means were separated using Tukey’s HSD. The level of significance was defined at *p* < 0.05. Experimental results are expressed as mean ± SE.

## Results

### Differential effects of LPS on NO, IL-6, and leptin production in murine macrophage and adipocytes

**Fig. 1:**
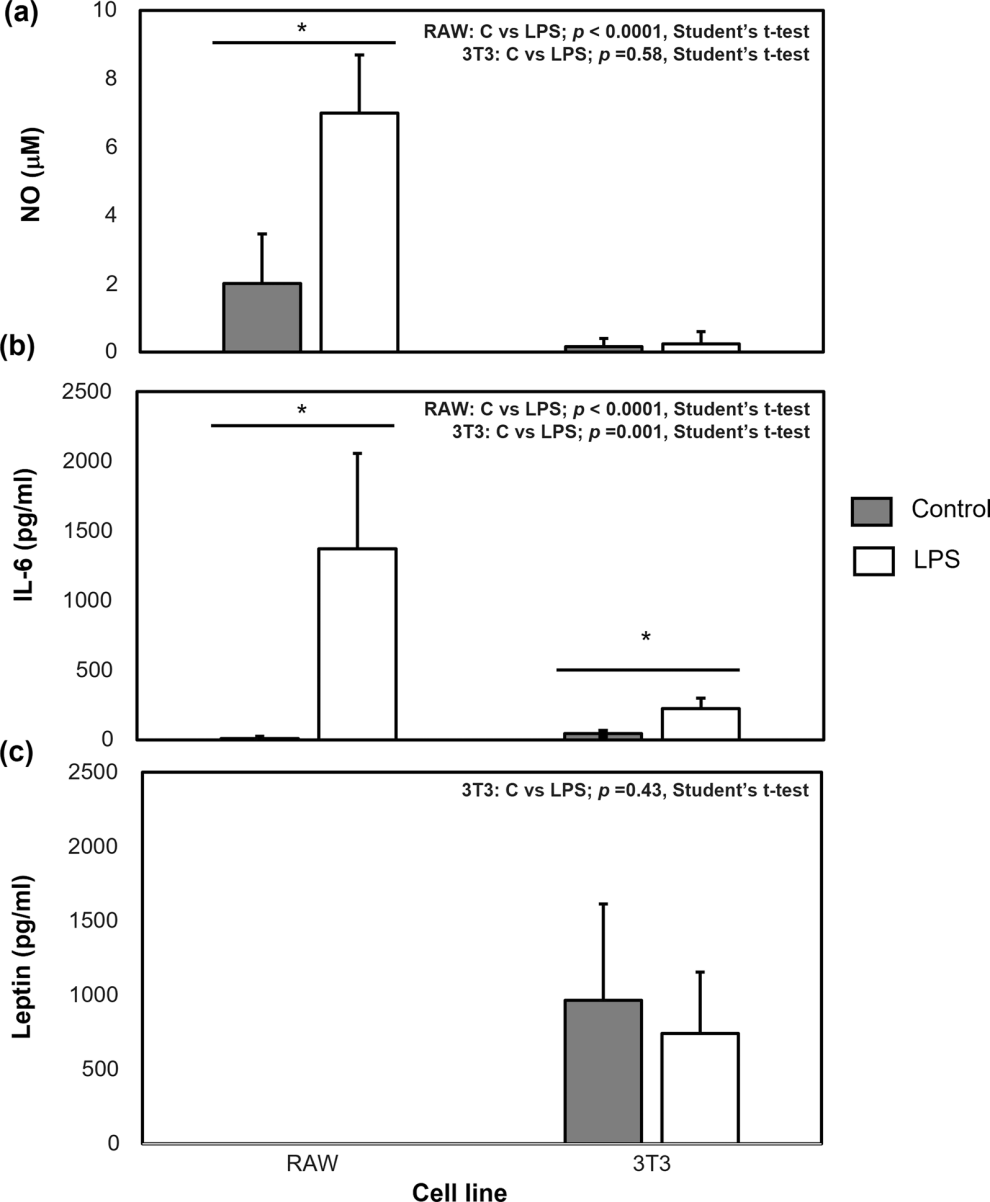
Nitric oxide (NO; μM, **a**), interleukin-6 (IL-6; pg/ml, **b**), and leptin (pg/ml, **c**) production by control and LPS-stimulated murine macrophages (RAW264.7) and adipocytes (3T3-L1). Adipokine concentrations in control and LPS-stimulated cells were compared using a Student’s *t* test. Data represent least squares means ± SEM of 4 independent experiments. There was no quantifiable leptin production by RAW 264.7 cells. Significant treatment differences are represented with an asterisk (*) (*p* < 0.05)

In order to determine the effect of an LPS challenge on macrophage activation (as measured by NO production), IL-6, and leptin production by murine macrophages and adipocytes, a series of control experiments were performed on the isolated cell lines. The RAW 264.7 macrophages were grown to 80–85% confluence, while 3T3-L1 cells were differentiated into the mature adipocyte phenotype, at which time LPS was added (0.01 μg/ml) and allowed to incubate for a period of 6 h. Lipopolysaccharide challenge resulted in an increase in macrophage activation, as measured by increased NO production (Fig. 1a; student’s *t* test, *p* < 0.0001), and IL-6 production (Fig. 1b; student’s *t* test, *p* < 0.0001), and increased IL-6 production by mature adipocytes (Fig. 1b; *t* test, *p* = 0.001). Nitric oxide production by mature adipocytes was negligible and did not differ between control and LPS-challenged cells (Fig. 1a; student’s *t* test, *p* = 0.58), while murine macrophages did not produce quantifiable amounts of leptin in either the control or LPS-challenged conditions (Fig. 1c). Adipocytes produced similar amounts of leptin in either the absence or presence of LPS (965.8 ± 648.2 vs 741.5 ± 413.2 pg/ml) (Fig. 1c; student’s *t* test, *p* = 0.43).

### Differences in the molecules quantified between transwell inserts and wells

Although the pores of the transwell insert membrane are large enough (0.4 μm) to allow the diffusion of molecules of varying sizes from areas of higher to lower concentration, we considered that at the time of sampling equilibrium might not have been reached, resulting in differences in microenvironment between the transwell inserts and the wells. At the end of the co-incubation experiments, we sampled cell-conditioned media from these two compartments and measured NO, IL-6, and leptin levels in them separately (Table 1). Higher amounts of the leptin adipokine were detected in adipocyte-containing wells (627.1 ± 47.5 pg/ml) compared to macrophage-containing transwell inserts (478.2 ± 47.5 pg/ml) (Table 1; matched pairs *t* test, *p* = 0.004). These findings are unsurprising as leptin is mostly, if not exclusively, produced by adipocytes, which were localized in the well compartment. The difference in leptin levels in wells and transwells indicates that if leptin is traveling across the transwell insert membrane, at the time of sampling levels of the molecule had not reached equilibrium. There were no significant differences in the amounts of NO and IL-6 quantified in the macrophage-containing transwell inserts compared to the adipocyte-containing wells (Table 1). The low levels of NO and IL-6 produced by control and LPS-challenged adipocytes (Fig. 1a, b) suggest that these molecules are produced by macrophages and traveling across the transwell membrane into the well compartment.

**Table 1:** Differences in NO, IL-6, and leptin amounts quantified between macrophage-containing transwell inserts and adipocyte-containing wells (average of all treatments)

Molecule	Average amount of molecule in transwell (macrophages)	Average amount of molecule in well (adipocytes)	Difference between transwell and well (matched pairs *t* test)
NO (mM)	7.2 ± 1.0	6.0 ± 1.0	*p* = 0.26
IL-6 (pg/ml)	1172.2 ± 122.0	1115.76 ± 122.0	*p* = 0.65
Leptin (pg/ml)	478.2 ± 47.5	627.1 ± 47.5	*p* = 0.004*

Matched pairs *t* tests with *p* < 0.05 indicate significant difference in amount of molecule measured in transwells and wells

### Effect of macrophage activation status on NO, IL-6, and leptin levels

We were interested in testing if different methods of macrophages activation resulted in quantifiable differences in NO and IL-6 production. Specifically, we challenged macrophages with LPS (6 h) at time of co-incubation with adipocytes (3T3 + RAW + LPS) or challenged them with LPS for 6 h before co-incubation with adipocytes and then either co-incubated the adipocytes with the previously LPS-challenged macrophages resuspended in fresh media (3T3 + StimRAW) or in their conditioned media (3T3 + stimRAW + CondMed).

**Fig. 2:**
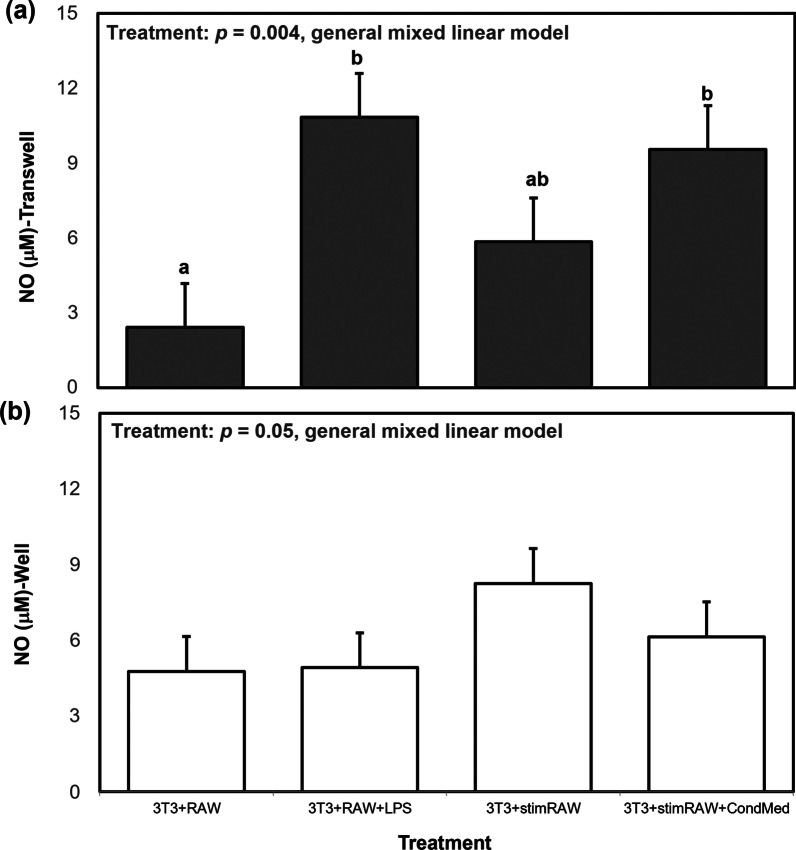
Mean nitric oxide (NO; μM) production for different co-incubation methods of 3T3-L1 + RAW cells transwells = **a**; wells = **b**). NO concentrations for the various co-incubation methods (“treatment”) were compared using a general mixed linear model, followed by post hoc Tukey–Kramer HSD. Data represent least squares means ± SEM of 4 independent experiments. Significant treatment differences are represented with different letters (*p* < 0.05)

**Fig. 3:**
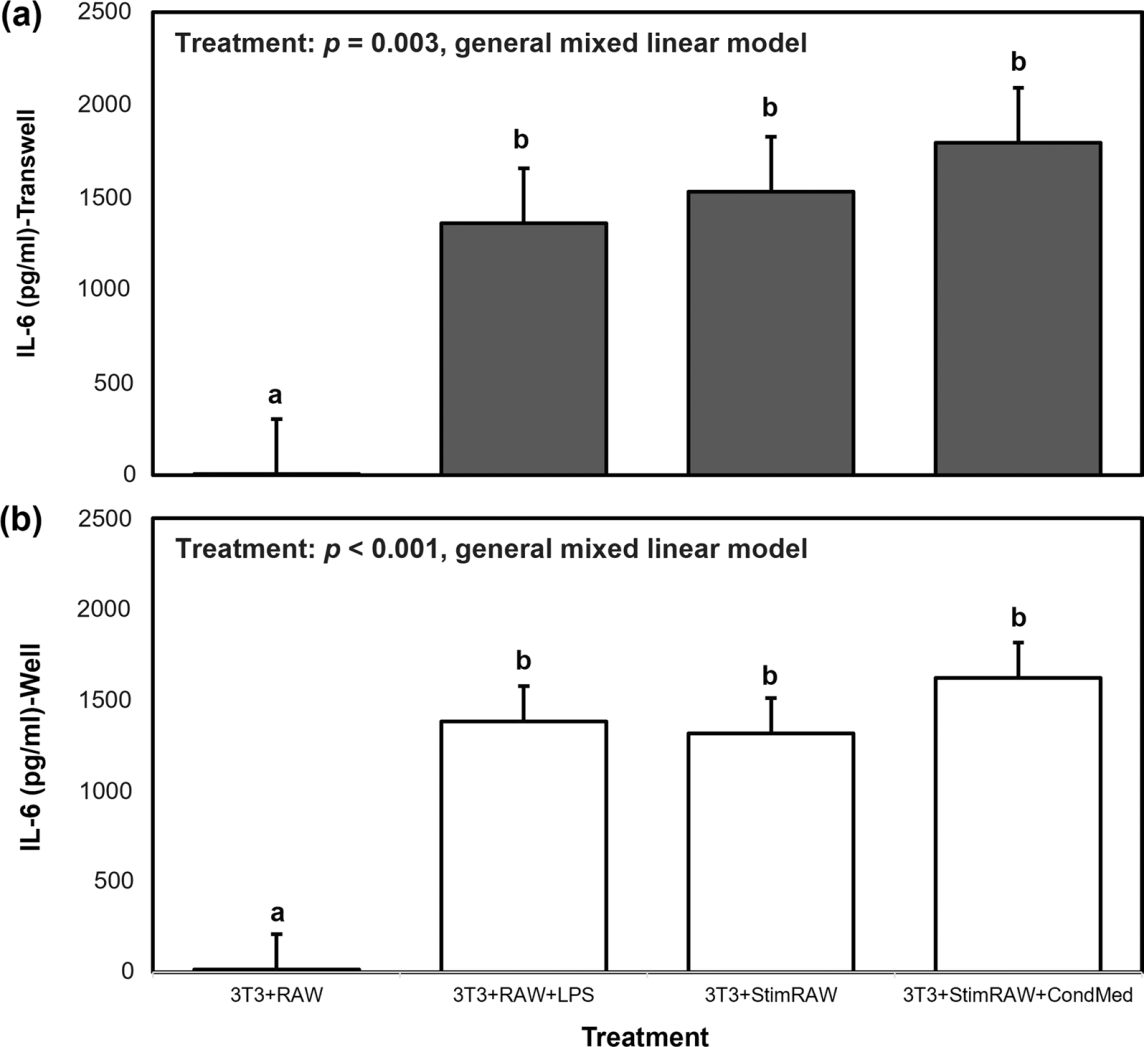
Mean interleukin-6 (IL-6; pg/ml) production for different co-incubation methods of 3T3-L1 + RAW cells (transwells = **a**; wells = **b**). IL-6 concentrations for the various co-incubation methods (“treatment”) were compared using a general mixed linear model, followed by post hoc Tukey–Kramer HSD. Data represent least squares means ± SEM of 4 independent experiments. Significant treatment differences are represented with different letters (*p* < 0.05)

As expected, the co-incubation methodologies with LPS-challenged macrophages (3T3 + RAW + LPS,) resulted in higher NO (Fig. 2a) and IL-6 (Fig. 3a, b) production compared to the control with unstimulated macrophages (3T3 + RAW) (general mixed linear model, *p* < 0.0001). When measuring adipokine levels in cell-conditioned media from transwell inserts, we observed no differences in levels of molecules that indicate macrophage activation (NO, IL-6) among the three co-incubation methodologies containing LPS-challenged macrophages, regardless of if the cells were activated before or during plating onto the transwell inserts, or if fresh or conditioned media was used (Figs. 2a, b and 3a, b; general mixed linear model, *p* < 0.001). Interestingly, in the well compartment there were no differences in NO levels among control and the LPS-challenged macrophage treatments (Fig. 2b; general mixed linear model, *p* = 0.05), while IL-6 levels were significantly higher in those treatments containing activated macrophages (Fig. 3b; general mixed linear model, *p* < 0.0001).

**Fig. 4:**
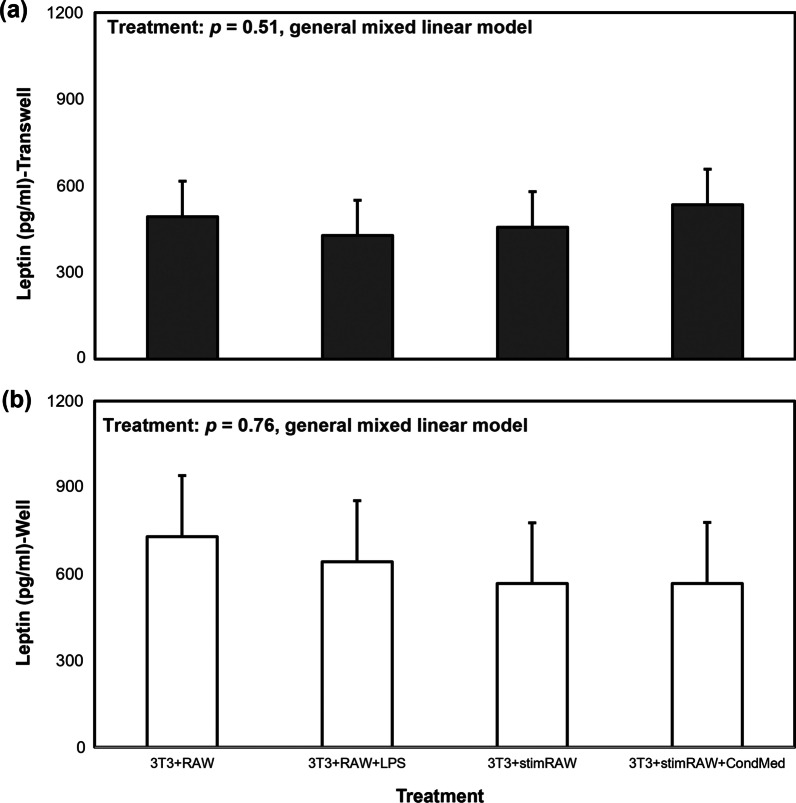
Mean leptin (pg/ml) production for different co-incubation methods of 3T3-L1 + RAW cells (transwells = **a**; wells = **b**). Leptin concentrations for the various co-incubation methods (“treatment”) were compared using a general mixed linear model, followed by post hoc Tukey–Kramer HSD. Data represent least squares means ± SEM of 4 independent experiments. There were no treatment effects on leptin levels in transwell inserts or wells

Higher amounts of the leptin adipokine were observed in adipocyte-containing wells compared to macrophage-containing transwells (Table 1), but macrophage activation status did not have an effect on leptin levels in either the macrophage-containing transwell inserts (Fig. 4a) or adipocyte-containing wells (Fig. 4b).

**Fig. 5:**
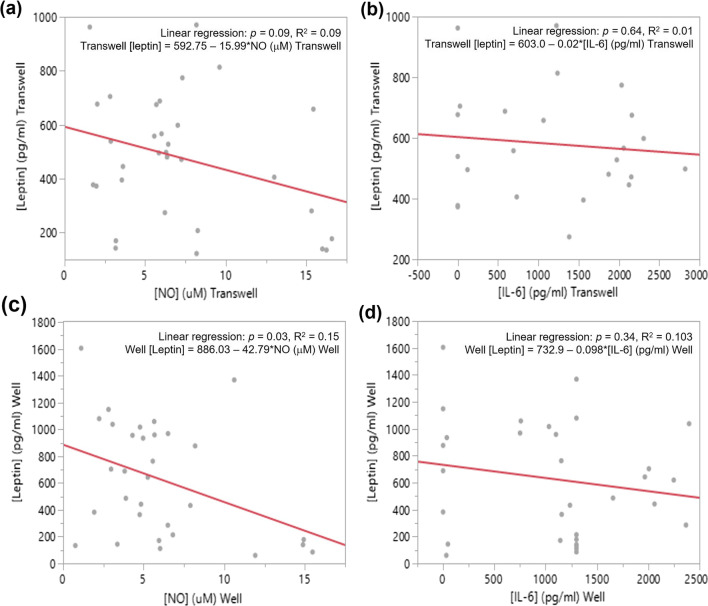
Relationship between NO and leptin and IL-6 and leptin concentrations measured in transwell inserts (**a**, **b**) and wells (**c**, **d**). We observed a trend for a negative relationship between NO and leptin levels measured in transwells (**a**; *p* = 0.09, linear regression), and a negative relationship between these two molecules in wells (**c**; *p* = 0.03, linear regression). There was no significant relationship between IL-6 and leptin levels in either transwells (**b**) or wells (**d**)

In transwell inserts, a trend for a negative linear relationship between levels of NO and leptin was observed (Fig. 5a; linear regression, *p* = 0.09), while a significant negative linear relationship between levels of NO and leptin was present in wells (Fig. 5c; linear regression, *p* = 0.03). There was no relationship between leptin and IL-6 levels in either wells or transwell inserts (Fig. 5b, d). It is important to highlight that the low coefficients of determinations (R^2^) for the relationships between NO and leptin indicate that there are high levels of variability in leptin levels that cannot be explained by NO levels.

## Discussion

There were significant differences in amounts of NO and leptin measured in transwells and wells. Higher NO levels were observed in cell-conditioned media collected from the macrophage-containing transwells, while the cell-conditioned media collected from adipocyte-containing wells contained higher levels of leptin. This is logical, as macrophages are the major contributors to NO while adipocytes are the major contributors to leptin in the cell-conditioned media. The assay used to determine NO levels measures concentrations of nitrite (NO^−^_2_), a small molecule (46 Da) that would be expected to travel through the pores of the transwell membrane (0.4 μm size). Diffusion of nitrite through the pores of the inserts would explain why there were quantifiable amounts of this molecule in the adipocyte-containing wells. Unno et al. (2006) reported that 3T3-L1 adipocytes treated with an interferon (IFN)-γ -LPS (10 ng/ml-5 mg/ml) mixture for 24 h had significantly increased expression of iNOS as well as of nitrite and nitrate released into culture medium, compared to untreated adipocytes. Dobashi et al. (2000) treated differentiated 3T3-L1 adipocytes with 1 μg/ml LPS for 48 h and saw no significant increase in NO production by adipocytes compared to non-LPS treated cells, suggesting that IFN-γ drives iNOS expression in 3T3-L1 cells to a larger extent than LPS. Our results support this idea, as in control experiments we did not observe a significant effect of LPS challenge on adipocyte-derived NO production (Fig. 1a). Taking these results into account, we hypothesize that in our co-incubation system the presence of NO in wells is due to diffusion of macrophage-derived NO from the transwell inserts into the wells.

Despite the differences in NO measured between transwells and wells, IL-6 levels in cell-conditioned media collected from these two compartments were similar. Adipocytes produce IL-6 under the action of LPS (Chirumbolo et al. 2014; Harlan et al. 2020), but it is unlikely that the lack of difference in IL-6 levels between the transwells and wells is due to adipocyte-secreted IL-6, since the control experiments showed that LPS-challenged adipocytes produced about a sixth the amount of IL-6 under the action of LPS compared to macrophages (RAW: 1371.8 ± 682.9; 3T3: 225.9 ± 73.4 pg/ml; Fig. 1b). We also suspect that LPS is not able to travel across the membrane of the transwell insert, where it was added to the macrophages. Although the small size of the LPS molecule (4.3 kDa) means that it is possible for it to travel across the transwell insert membrane, its heterogeneous nature can result in aggregates of varying sizes. These aggregates can range in size from 1000 to 4000 kDa (Jann et al. 1975), which would be too large to travel through the 0.4 mm pore size of the membrane. It is more plausible that IL-6, with a 21 kDa size, is small enough to diffuse across the transwell membrane which would allow for equilibrium to be reached between the macrophage and adipocyte-containing compartments. The possibility that IL-6 production by macrophages could start leveling off before that of NO would explain the difference in NO levels observed between transwells and wells, and the lack of difference observed for IL-6. We should consider that if LPS is traveling across the transwell membrane it could be having a more pronounced effect on the co-incubated adipocytes than demonstrated in the control experiments that tested the effects of this endotoxin on the isolated cell lines. Yamashita et al. (2007) found that IL-6 production was markedly up-regulated in adipocytes co-cultured with macrophages in the presence of LPS, compared to stimulating each cell line separately with the endotoxin. It is unlikely that in our system the IL-6 quantified in the wells is derived from the adipocytes, as levels of this adipokine are similar in cell-conditioned media from wells containing adipocytes co-incubated with macrophages in the presence of LPS (3T3 + RAW + LPS) and wells containing adipocytes co-incubated with previously LPS-activated macrophages that were washed and resuspended in fresh medium (removing LPS) at the time of co-incubation (3T3 + StimRAW).

The adipocyte-specific adipokine leptin was found in higher quantities in the wells, where the adipocytes resided. Temporal differences in expression of the leptin gene and production of the protein could explain the difference in transwell vs well concentrations. The leptin molecule, at 16 kDa, is smaller than IL-6, which would allow it to travel across the transwell membrane. If leptin secretion by the adipocytes is occurring 24 h post-co-incubation, this would explain why levels of this adipokine are different between transwells and wells during collection of cell-conditioned media. Our data show that there is a basal leptin production by the adipocytes that is not dependent on LPS stimulation, as adipocytes exposed to the control treatment secreted the same amount of this adipokine as adipocytes co-incubated with LPS. Previous reports have found that both physiological and pathological levels of leptin do not induce the expression of IL-6 in murine macrophages, but that it augments the effect of LPS in inducing IL-6 expression by priming macrophages to be more responsive to this endotoxin and that this synergistic effect is mediated by interleukin-receptor-associated kinase (IRAK)-1 (Vaughan and Li 2010). It is difficult to extrapolate these results to our own, since in our system we did not observe a difference in IL-6 levels between control experiments (macrophages activated with LPS) and macrophages in the co-incubation system which were activated with LPS and exposed to adipocyte-derived leptin. Furthermore, activation status of the macrophages did not affect leptin production although there appeared to be a weak relationship between leptin and NO levels, with leptin levels decreasing as NO levels increased. These results match what was observed by Unno et al. (2006), who treated differentiated 3T3-L1 cells with an interferon (INF)-γ-LPS mixture and observed a significant induction of iNOS and decrease in leptin at both the protein and mRNA levels. We need to take into account that despite the observed significant relationship between these molecules, the low R^2^ values indicate that there are other factors influencing this relationship. Use of INF-γ, in addition to LPS, in these co-incubation experiments could help clarify the relationship between leptin and NO in adipocytes. We also need to consider that the NO present in our system is mostly derived from macrophages, while in Unno’s system NO was produced autocrinally by the adipocytes or due to the synergistic effect of LPS and INF-γ. We hypothesize that nitric oxide might have differential autocrine and paracrine effects on leptin protein and gene expression in adipocytes. This is a question that warrants further investigation in order to better understand the effects macrophage-derived NO could potentially have on mature adipocytes in this co-incubation system.

As a methodology, co-incubation of activated macrophages and fully differentiated adipocytes can help answer questions about macrophage-adipocyte interactions in AT and provide insights into how to blunt the inflammatory loop observed in obese AT. Research on this subject suggests that monocyte/macrophage recruitment into obese AT is an early contributor to this loop by virtue of macrophages having a bigger role in the secretion of inflammatory adipokines compared to adipocytes (Bai and Sun 2015; Fain 2010). The polarization of macrophages toward the M1 phenotype as they arrive in obese AT is presumably directed by the microenvironment encountered by the macrophages, which is mostly set by the adipocytes residing in the tissue (Fain 2010; Makki et al. 2013; Lindhorst et al. 2021). We wanted to examine if activation of macrophages before or during plating affected secretion of inflammatory molecules, like NO and IL-6, by these cells and if activation status of the macrophages had an effect on production of the adipocyte-specific inflammatory adipokine leptin. Results from these experiments can help clarify the role of macrophage-derived adipokines on the initiation of the macrophage-adipocyte inflammatory loop observed in obese adipose tissue.

We did not observe significant difference in NO or IL-6 production from macrophages regardless of if LPS was added before plating or during plating onto transwells. As expected, increased NO production by the macrophages, a sign of macrophage activation, was accompanied by increased production of IL-6. Release of IL-6 by macrophages suggests that these macrophages are taking on an M1 phenotype, which is expected during LPS activation (Orecchioni et al. 2019). Although the three methodologies of macrophage activation tested did not result in significant differences in NO and IL-6 production, it is important to note that at the time of cell-conditioned media collection, the media from macrophages plated in conditioned media (3T3 + StimRAW + CondMed) contained secreted products for a 30 h time period while macrophages in fresh media (3T3 + StimRAW) or with LPS added at time of plating (3T3 + RAW + LPS) contained 24 h of secreted products (Figs. 2, 3). Levels of NO produced by activated macrophages plated in fresh media were similar to levels of NO produced by unstimulated macrophages, suggesting that in this specific cell culture system these cells produced basal amounts of NO without LPS activation. This could be explained by activation of the macrophages as they are transferred from their original culture system (6-well plate) to the transwells via the cell scraping method. On the other hand, quantified IL-6 levels were significantly higher in cell-conditioned media collected from activated macrophages plated in fresh media compared to unstimulated macrophages, indicating that in this cell culture methodology challenging RAW 264.7 cells with LPS has a more pronounced and/or prolonged effect on production and release of IL-6 compared to NO.

## Conclusions

The presence of LPS-stimulated macrophages in the co-incubation system did not affect leptin release by the mature adipocytes, as the adipocytes produced similar leptin levels when co-incubated with activated macrophages (3T3 + RAW + LPS, 3T3 + StimRAW, 3T3 + StimRAW + CondMed) as when co-incubated with unstimulated macrophages (3T3 + RAW) (Fig. 4). Our results also highlight the importance of sampling and analyzing the macrophage and adipocyte-containing microenvironments (transwells and wells, respectively) separately, in order to detect the subtle signaling dynamics that are important in the paracrine conversation occurring between these cell types. The methodologies presented here can be adopted for the study of macrophage-adipocyte interactions, including cellular communication, chemotaxis studies, and effects of macrophage-derived molecules on adipocyte differentiation and mitochondrial function, among other research areas. Constant et al. (2008) stated that the ERK 1/2-driven antiadipogenic effect of macrophage cell-conditioned media on adipocytes occurred during the first 2 days of the 8-day adipocyte differentiation period. The co-incubation protocols we have developed can be modified to test cellular communication between these two cell types at different time points, allowing for further analyses of temporal interactions. Transwell inserts with larger pore sizes (3–5 μm) can be used for migration and chemotaxis studies that can help answer questions about macrophages recruitment into obese AT, which appears to be one of the early steps in setting up the macrophage-adipocyte inflammatory loop.

## Supplementary Information

**Additional file 1: Fig. S1**. Undifferentiated 3T3-L1 fibroblasts (panel a, left) and fully differentiated 3T3-L1 adipocytes with lipid droplets stained with oil red O (panel a, right) at 40 × magnification. Differentiated adipocytes contained significantly more lipid droplets as quantified via spectrophotometric analysis (absorbance at 520 nm) of dissolved oil red O stain compared to undifferentiated fibroblasts (panel b) (student’s *t* test). Data represent least squares means ± SEM of 3 independent experiments. Significant treatment differences are represented with an asterisk (*) (*p* < 0.0001).

**Additional file 2: Fig. S2**. Mean interleukin-6 (IL-6; pg/ml; panel a) and nitric oxide (NO; mM; panel b) production for different lipopolysaccharide (LPS) doses (0.01, 0.1 μg/ml) and incubation times (3 h, 6 h, 24 h) in RAW 264.7 cells. IL-6 and NO concentrations were compared using a two-way ANOVA, followed by post hoc Tukey–Kramer HSD. Data represent least squares means ± SEM of 2 independent experiments. Significant treatment differences are represented with different letters (*p* < 0.05).

## Data Availability

The data that support the findings of this study are openly available in DRYAD at 10.5061/dryad.c59zw3r7w.

## Abbreviations

AT: Adipose tissue
IL: Interleukin
TNF-α: Tumor necrosis factor-alpha
MCP-1: Monocyte chemoattractant protein-1
NO: Nitric oxide
iNOS: Inducible nitric oxide synthase
LPS: Lipopolysaccharide
DMEM: Dulbecco’s modified Eagle’s medium
PBS: Phosphate buffered saline
FBS: FETAL bovine serum
DEX: Dexamethasone
IBMX: 3-Isobutyl-1-methylxanthine
NED: N-1-naphthylethylenediamine
IRAK-1: Interleukin receptor-associated kinase
INF: Interferon
