# Supplementary figures and images for: Effects of co-incubation of LPS-stimulated RAW 264.7 macrophages on leptin production by 3T3-L1 adipocytes: a method for co-incubating distinct adipose tissue cell lines
Source: Bull Natl Res Cent. 2022 Mar 7;46(1):57. doi: 10.1186/s42269-022-00747-7 (PMC8899443; doi:10.1186/s42269-022-00747-7)

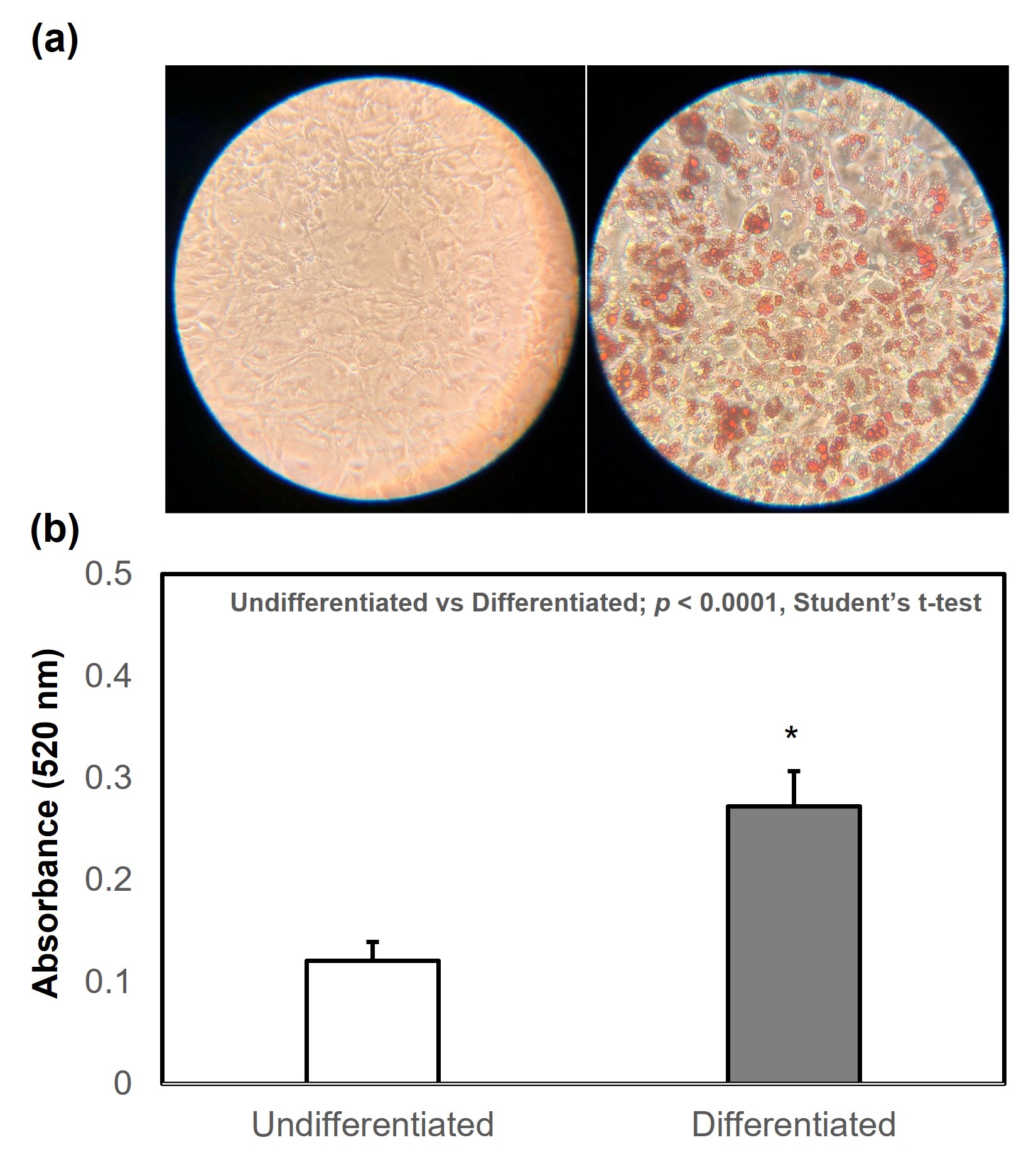

Supplement: Supplementary file 1 — Additional file 1: Fig. S1. Undifferentiated 3T3-L1 fibroblasts (panel a, left) and fully differentiated 3T3-L1 adipocytes with lipid droplets stained with oil red O (panel a, right) at 40 × magnification. Differentiated adipocytes contained significantly more lipid droplets as quantified via spectrophotometric analysis (absorbance at 520 nm) of dissolved oil red O stain compared to undifferentiated fibroblasts (panel b) (student’s t test). Data represent least squares means ± SEM of 3 independent experiments. Significant treatment differences are represented with an asterisk (*) (p < 0.0001). [file 42269_2022_747_MOESM1_ESM.jpg]

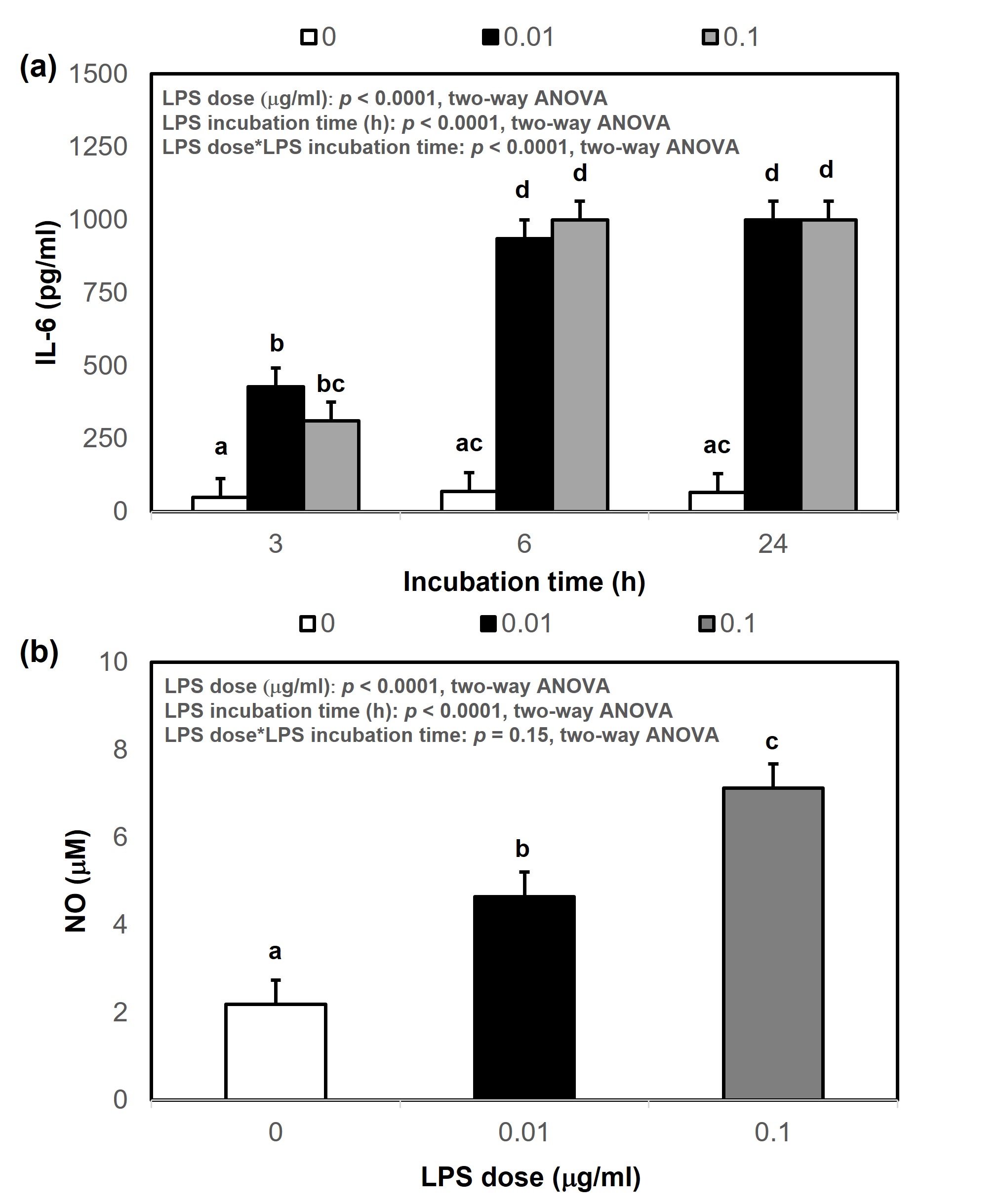

Supplement: Supplementary file 2 — Additional file 2: Fig. S2. Mean interleukin-6 (IL-6; pg/ml; panel a) and nitric oxide (NO; mM; panel b) production for different lipopolysaccharide (LPS) doses (0.01, 0.1 μg/ml) and incubation times (3 h, 6 h, 24 h) in RAW 264.7 cells. IL-6 and NO concentrations were compared using a two-way ANOVA, followed by post hoc Tukey–Kramer HSD. Data represent least squares means ± SEM of 2 independent experiments. Significant treatment differences are represented with different letters (p < 0.05). [file 42269_2022_747_MOESM2_ESM.jpg]
